# The Pharmaceutical Innovativeness Index: Supporting Value-Based Economic Regulation of Innovative Medicines

**DOI:** 10.3390/jmahp13040060

**Published:** 2025-12-08

**Authors:** Ludmila Peres Gargano, Marcus Carvalho Borin, Wallace Mateus Prata, Juliana Alvares-Teodoro, Francisco de Assis Acurcio, Roberto F. Iunes, Augusto Afonso Guerra

**Affiliations:** 1 Department of Social Pharmacy, School of Pharmacy, Federal University of Minas Gerais (UFMG), Av. Presidente Antônio Carlos, 6627, Belo Horizonte 31270-901, MG, Brazil; 2 SUS Collaborating Center for Technology Assessment and Excellence in Health, School of Pharmacy, Federal University of Minas Gerais (UFMG), Av. Presidente Antônio Carlos, 6627, Belo Horizonte 31270-901, MG, Brazil; 3 Hospital Alemão Oswaldo Cruz (HAOC), Health Technology Assessment Unit (UATS), Av. Paulista, 500, Bela Vista, Sao Paulo 01310-000, SP, Brazil; 4 Fundação Ezequiel Dias (FUNED)—Diretoria de Pesquisa e Desenvolvimento (DPD), Rua Conde Pereira Carneiro, 80, Gameleira, Belo Horizonte 30510-010, MG, Brazil; 5 World Bank, 1818 H Street, N.W., Washington, DC 20433, USA

**Keywords:** pharmaceutical pricing, value-based pricing, innovation, health technology assessment, regulatory policy

## Abstract

The regulation of new medicine prices must balance financial sustainability with equitable access to innovation. Value-Based Pricing (VBP) strategies seek to align drug prices with their clinical and societal impact. The Pharmaceutical Innovativeness Index (PII) is a transparent and reproducible tool proposed to assess the degree of innovativeness of new medicines, with potential to support pricing decisions within economic regulation frameworks. An exploratory qualitative study was conducted through a focus group study was conducted with experts in health economics and pharmaceutical regulation to evaluate the applicability of the PII and to discuss key domains relevant to the assessment of pharmaceutical innovation. Responses were collected anonymously using an interactive digital platform and analyzed through inductive thematic content analysis. Based on these findings, the research team developed a conceptual pricing model integrating the PII with additional value-based criteria. Two hypothetical case studies were created to explore its practical feasibility. Participants identified Added Therapeutic Value (ATV) and Unmet Therapeutic Need (UTN) as the most relevant domains, while Methodological Quality (MQ) and Study Design (SD) were also recognized as essential to ensure rigor and reduce bias. The PII scores showed strong alignment with expert judgment in the illustrative case studies. The proposed model incorporates international best practices—such as the efficiency frontier approach—and additional dimensions including safety and incremental innovation. Overall, the PII demonstrated potential to enhance transparency, consistency, and regulatory efficiency in drug pricing decisions in Brazil. However, it should currently be regarded as an exploratory framework requiring further empirical validation and regulatory adaptation before implementation.

## 1. Introduction

The pricing regulation of new medicines is an increasing challenge for healthcare systems and the pharmaceutical industry, as it requires balancing financial sustainability, access to innovation, and incentives for research and development (R&D) [1,2]. Different countries have adopted regulatory models that reflect their socioeconomic realities and health priorities [3]. Value-Based Pricing (VBP) has emerged as an alternative to traditional regulation, incorporating clinical efficacy, quality of life, and broader social impact into the recognition of a medicine’s value [4,5,6]. Frameworks currently adopted in countries like France, Italy and Germany provide useful benchmarks on how therapeutic value can inform pricing and reimbursement decisions. In France, the Haute Autorité de Santé (HAS) evaluates absolute and relative therapeutic benefit, linking these classifications to pricing and reimbursement level [7]. In Italy, the Agenzia Italiana del Farmaco (AIFA) assesses innovation by combining Added Therapeutic Value (ATV), Unmet Therapeutic Need (UTN), and evidence quality [8]. Germany, in turn, applies efficiency frontier (EF) that link incremental clinical benefit to acceptable cost thresholds [9]. While these frameworks represent mature and robust approaches, their design and implementation are deeply tied to specific national contexts, limiting their direct applicability to other countries. Nonetheless, they offer a valuable foundation for developing models adapted to the Brazilian regulatory environment.

In Brazil, the Drug Market Regulation Chamber (CMED) [10] oversees pricing policy to promote medicine availability and sector competitiveness. However, since Resolution No. 2/2004, the lack of updates in pricing criteria has created regulatory gaps, particularly in evaluating advanced therapies, orphan drugs, and highly innovative medicines. Key limitations include the absence of clear criteria for drugs without international references or comparators and insufficient recognition of incremental innovation [10,11]. As a result, prices often fail to reflect therapeutic value, threatening system sustainability and discouraging genuine innovation.

The concept of valuable pharmaceutical innovation lacks universal consensus [12], but it is generally associated with the ability of a health technology to improve survival, reduce disease burden, and enhance quality of life [13,14,15,16]. Building on this understanding, the Pharmaceutical Innovativeness Index (PII) was proposed as a transparent and reproducible tool to assess innovativeness at market entry, integrating both clinical and methodological parameters [13]. The PII comprises four domains: two clinical—Added ATV and UTN—and two methodological—Methodological Quality (MQ) and Study Design (SD)—to ensure that innovation assessment reflects both therapeutic relevance and the robustness of supporting evidence. Similarly to other international frameworks, the PII seeks to classify the degree of innovation based on therapeutic value and evidence quality, but with the specific purpose of enhancing transparency and reproducibility in regulatory evaluations, while also provides a quantitative score that summarizes the overall level of innovativeness, allowing for objective comparison across medicines and therapeutic areas [13].

Building on these foundations, the present study proposes a combined strategy: first, adapting the PII framework in line with the Italian approach to classifying pharmaceutical innovativeness [17]; and second, incorporating elements of EF to establish price benchmarks when no therapeutic comparators are available. By integrating these two approaches, this work proposes a structured model for applying the PII in Brazil, aiming to align prices with therapeutic and social value, enhance transparency in pricing decisions, and ensure the financial sustainability of the health system. The study also examines the perceptions of regulatory experts regarding the feasibility of implementing the PII, offering insights into how international experiences can be adapted to support value-based economic regulation in emerging contexts.

This study does not aim to present a finalized or validated instrument, but rather to explore the feasibility of applying the PII within a regulatory context. The work adopts a methodological and conceptual perspective, combining expert input and international benchmarking to propose a pilot model for value-based drug pricing in Brazil. Therefore, the study should be interpreted as an exploratory framework intended to inform future policy development and empirical testing, rather than as a definitive guideline.

## 2. Materials and Methods

This study was designed as a methodological and exploratory analysis to assess the applicability of the PII to the Brazilian regulatory framework for drug pricing. The aim was not to redevelop or revalidate the PII, but to explore its potential use as a decision-support tool for value-based economic regulation. The study combined two complementary stages: (1) a qualitative focus group with experts in health economics and pharmaceutical regulation, and (2) the conceptual development of a pricing model adapted to the Brazilian context.

### 2.1. Focus Group: PII as a Tool to Support Economic Regulation in Brazil

**Figure 1:**
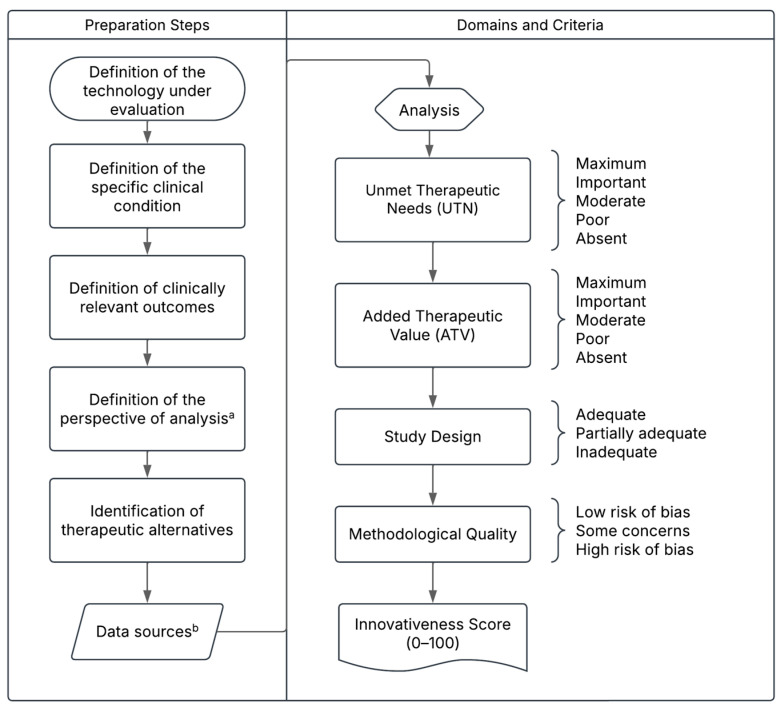
Proposed Structure of the Pharmaceutical Innovativeness Index (PII). Source: authors’ elaboration. Notes: a. The analytical perspective for pricing new medicines refers to the point of market entry. In cases of price adjustments over time, the temporal perspective may shift to the introduction of a new treatment for the same clinical indication, which would justify a reassessment of the prices of existing technologies. b. Data sources may include the drug’s dossier and pivotal clinical trials for market entry pricing, or real-world evidence in the case of price reassessment for technologies already available on the market.

An analytical and exploratory focus group was conducted to collect expert opinions on the applicability of the PII as a tool (Figure 1 and briefly described in Appendix A) to support economic regulation in Brazil. The session took place during a national Health Technology Assessment (HTA) congress held in Brazil, although it was organized independently and was not part of the official congress program. Professionals attending the event who had previous experience or institutional involvement in drug pricing and regulation were invited to participate, ensuring the inclusion of perspectives from government agencies, academia, and regulatory contexts.

The focus group was moderated by a researcher and followed a semi-structured discussion format. The session began with a brief presentation of the PII framework, including its domains and examples of application in real case studies. Following this introduction, participants were asked to share their perceptions of the tool’s relevance, feasibility, and potential role in pricing and reimbursement processes.

To promote open dialog and minimize response bias, opinions were collected anonymously through the Mentimeter^®^ (Mentimeter AB, Stockholm, Sweden) online platform, which allowed participants to submit their responses via personal devices without identification. This format encouraged spontaneous input and reduced the influence of hierarchical or institutional dynamics often present in group discussions.

The questions addressed included:

- The overall adequacy of the PII as a framework for assessing pharmaceutical innovation;
- The perceived relevance of each domain (ATV, UTN, MQ, and SD) in the context of pricing regulation;
- The potential need for additional domains or adaptations to better fit the Brazilian regulatory environment.

Participant academic backgrounds were recorded, but no personal data such as age or gender were collected.

The qualitative data collected through Mentimeter^®^ were analyzed using inductive thematic content analysis, in which themes and categories were identified directly from participants’ responses without predefined codes. The analysis was conducted manually by one researcher and subsequently discussed within the research team to ensure interpretative consistency. Responses were grouped into thematic categories reflecting key aspects of the Pharmaceutical Innovativeness Index (PII), including therapeutic value, unmet need, methodological quality, safety, and incremental innovation. Descriptive statistics (mean and standard deviation) were also calculated for the quantitative questions using Microsoft Excel^®^ (Office 365, Microsoft Corporation, Redmond, WA, USA).

Participation was voluntary and anonymous, and no identifying information was collected. The study followed the ethical principles established by the Brazilian National Health Council Resolution No. 510/2016, which exempts opinion surveys without sensitive or identifiable data from formal ethics committee review.

### 2.2. Proposed Model for Economic Regulation and Use Cases: Simulating the Use of the PII in the Pricing Process of Pharmaceutical Innovations

Following the focus group discussions, the research team conducted a structured synthesis of the findings to inform the development of a proposed economic regulation model for pharmaceutical pricing in Brazil. The model-building process was guided by the principles of VBP and incorporated international experiences from countries where therapeutic benefit directly influences pricing and reimbursement decisions.

The research team—comprising professionals with experience in HTA, economic evaluation, and regulatory policy—met in a series of analytical sessions to integrate qualitative feedback from the focus group with evidence from the literature and the Brazilian regulatory framework. The objective was to design a model that retained the conceptual integrity of the PII while adapting it to the national context and institutional constraints.

The proposed model combines the structure of the PII, aligned with the Italian approach to classifying innovation, with EF principles used in Germany to anchor pricing. These elements were adapted to fit within the operational framework of CMED, which defines market entry prices for new medicines.

The model establishes a stepwise process in which PII scores and economic modeling jointly inform pricing decisions and subsequent reassessments based on real-world evidence. It also incorporates provisions for recognizing incremental innovation attributes—such as adherence, safety, and convenience—when supported by robust data. The final proposal was refined through iterative discussions among the authors to ensure methodological consistency, feasibility, and alignment with international best practices.

The proposed framework was then tested in two illustrative case studies designed as hypothetical but realistic scenarios inspired by actual therapeutic contexts. The first case represented a continuous-use therapy for a chronic non-communicable disease with existing treatment alternatives, while the second simulated a one-time therapy for a rare condition with no specific treatment options, currently managed with palliative care. The parameters for these use cases were informed by focus group discussions and publicly available regulatory and clinical data. These scenarios served to demonstrate the practical application of the model to distinct clinical and regulatory settings. Detailed results of the simulations are provided in the Appendix A and Appendix B.

## 3. Results

### 3.1. General Perceptions from the Focus Group

A total of 27 professionals in HTA and economic regulation participated in the focus group and completed the opinion survey. Participants had academic backgrounds primarily in pharmacy (59.3%), followed by economics (11.1%), law (11.1%). Overall, participants considered the PII to be an appropriate tool for evaluating pharmaceutical innovation. On a scale from 0 (inadequate) to 10 (adequate), the average score was 7.77 (standard deviation, sd 1.42).

Regarding the relevance of the PII domains in general innovativeness assessment (not restricted to economic regulation), ATV and UTN received the highest scores—9.26 (sd 1.58) and 8.41 (sd 1.93), respectively—indicating strong consensus. Methodological domains, MQ and SD, received average scores of 7.67 (sd 2.20) and 8.00 (sd 2.37). “Other” domains scored a mean of 5.30 (sd 4.03), with 66.7% (*n *= 18) assigning them a non-zero value, suggesting perceived relevance beyond the four original PII domains.

When asked whether the PII could support decision-making in economic regulation, all respondents of this question (*n *= 24) agreed, though 75.0% believed the tool would require adjustments for this context.

To explore potential modifications, participants were asked to redistribute 100 points among the four PII domains based on their perceived relevance, simulating alternative weightings for both general innovativeness assessment and economic regulation contexts. The relative importance attributed to each domain was then converted into proportional weights totaling 100 points—33.2 for ATV, 29.0 for UTN, 18.0 for SD, and 19.8 for MQ. These weights were subsequently applied in the case studies to calculate the final PII scores for each hypothetical technology. Results are summarized in Table 1.

In the open-text responses, participants proposed several additional dimensions that should be considered in the assessment of pharmaceutical innovativeness. The most frequently mentioned aspects were incremental innovation, safety, and market or patent-related factors, as well as a few broader considerations related to accessibility and real-world performance.

Incremental innovation was highlighted through references to improvements in formulation, route of administration, dosage, and ease of use, which were perceived as relevant for enhancing treatment adherence and persistence. Participants emphasized that such refinements, although often considered minor from a regulatory standpoint, can produce meaningful gains in real-world outcomes by simplifying use and improving patient experience.

Safety was also identified as a critical factor in evaluating innovation. Respondents noted that therapeutic advances should not be limited to efficacy improvements, as reductions in adverse events or better tolerability can represent substantial value for both patients and health systems. Some participants also pointed the balance between safety and efficacy, suggesting that innovations achieving safety gains without compromising clinical effectiveness should be appropriately recognized.

Other comments addressed broader contextual elements, including the development of the national pharmaceutical industry, patent status, and place of manufacturing, all viewed as potentially relevant to the strategic value of innovation. Participants suggested that pricing regulation could include incentives for locally developed technologies, a prioritizing national innovation could reduce import dependence, foster technological capacity, and stimulate R&D investment.

A few respondents mentioned the importance of cost-effectiveness, post-marketing performance, and the accessibility of innovations for specific patient groups. These reflections suggest an interest in expanding the concept of innovativeness beyond clinical outcomes to encompass societal, industrial, and other patient-centered dimensions.

One specific comment addressed process innovation in pharmaceutical manufacturing. While widely acknowledged as essential for efficiency and cost reduction, its impact is rarely factored into pricing decisions, as it does not directly translate into clinical outcomes.

The regulatory challenge lies in designing a model that recognizes these additional dimensions without compromising transparency or predictability. In this context, the next section presents a proposed model for economic regulation in Brazil that integrates the core domains of the PII (UTN, ATV, MQ, and SD) alongside additional those additional factors discussed on the focus group to ensure they are appropriately valued while maintaining the financial sustainability of the health system.

### 3.2. Proposed Model for the Economic Regulation of Health Technologies

**Figure 2:**
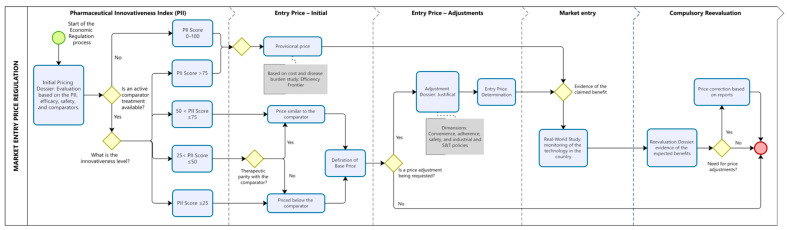
Proposed Model for the Economic Regulation of Pharmaceutical Innovations. The figure illustrates the sequential steps for defining the entry price of new pharmaceuticals using the PII to stratify innovativeness levels and guide pricing decisions. Blue rounded rectangles represent procedural steps; yellow diamonds indicate decision points; and grey rectangles highlight criteria used to justify price adjustments. The dotted vertical lines demarcate the four phases of regulation (initial assessment, initial price, price adjustment, market entry, and compulsory reevaluation). The red circle indicates the end point of the regulatory cycle. Note: This figure is conceptual and does not depict real regulatory procedures.

The proposed pricing regulation model (Figure 2) establishes two distinct pathways for determining market entry prices, depending on whether therapeutic comparators are available in Brazil. This approach aims to balance incentives for innovation, financial sustainability of the healthcare system, and alignment of prices with the clinical and economic value of technologies. In both cases, the process begins with the submission of a dossier by the company, which must include first, an assessment of the technology using the PII, classifying its degree of innovativeness, and second, an economic justification for the proposed price, based on EF and economic modeling studies (when applicable).

Based on the PII score and the availability of therapeutic alternatives, pricing will follow one of two regulatory pathways.

#### 3.2.1. Regulatory Pathway for Technologies Without Available Comparators

For technologies with no existing therapeutic alternatives—such as treatments for rare diseases or first-in-class therapies—market entry pricing should be based on economic modeling. In these cases, the comparative assessment must rely on the best available supportive care in the country, enabling estimation of incremental clinical and economic benefit relative to current management practices. The provisional base price should be grounded in this modeling exercise, supported by cost-of-illness studies and health outcome projections.

Alternatively, when an active comparator is not available, the PII may be applied comparatively across technologies from similar innovation profiles, such as gene or cell therapies, or medicines for rare diseases with comparable clinical characteristics. The PII score provides a quantitative and standardized measure of innovativeness, allowing the construction of an EF that links therapeutic value to price. In this framework, the PII score is plotted on the *x*-axis as a proxy for the degree of innovativeness, while the corresponding regulated or estimated price is plotted on the *y*-axis.

Each point on the EF represents the relationship between innovation level and market value for comparable technologies already approved. The expected price for the new technology is then inferred from the position it occupies along the frontier, reflecting its proportional innovativeness in relation to existing options. This method promotes consistency and fairness in pricing, particularly when traditional cost-effectiveness thresholds cannot be directly applied due to limited evidence.

**Figure 3:**
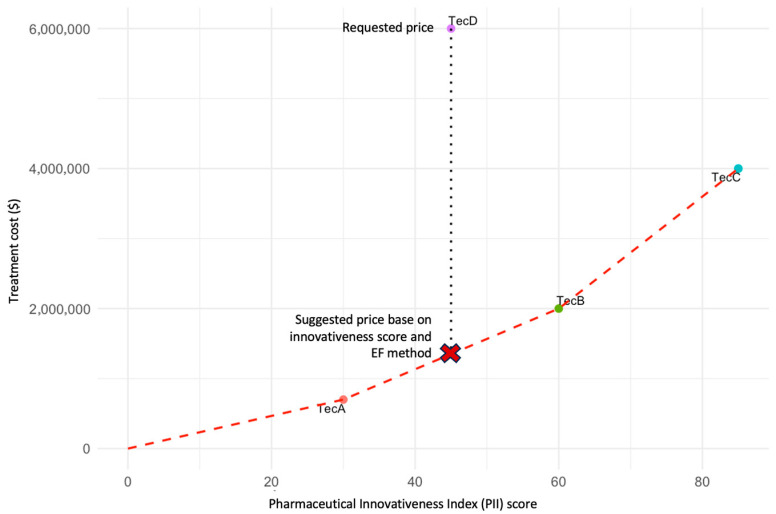
EF based on the PII. The figure illustrates an EF constructed using the PII score (*x*-axis) and the regulated price (*y*-axis) for three existing technologies (TecA, TecB, and TecC) and a new technology (D) under evaluation. Each point represents the relationship between the degree of innovativeness and the approved market price. The red dashed line represents the efficiency frontier connecting existing technologies. The vertical dotted line indicates the price requested by the manufacturer for TecD. The red “X” denotes the suggested price for TecD based on its PII score and the EF method. The proposed price for technology D should correspond to the position on the EF between technologies A and B, reflecting its relative innovativeness as measured by the PII. This approach ensures consistency and equity in price determination by linking incremental innovation to economic value. Note: This figure is illustrative and does not depict real data. It was developed to demonstrate the conceptual use of the PII as a quantitative metric within efficiency frontier modeling.

This conceptual exercise is illustrated in Figure 3, which presents three existing technologies (A, B, and C) and a new technology (D) under evaluation. The price of technology D is proposed to correspond to the position on the EF between technologies A and B, ensuring alignment between incremental innovation and economic value. This illustration is intended solely to demonstrate the conceptual use of the PII in defining efficiency frontiers. A forthcoming publication will provide a detailed methodological description and real-world case studies linking PII scoring to EF modeling.

As a condition for maintaining the established price, the manufacturer must commit to conducting real-world evidence studies in the country. These data will support a mandatory price reassessment two to three years after market entry. If the expected clinical and economic benefits are confirmed, the price may be maintained; otherwise, downward adjustments may be required to ensure alignment with the technology’s actual value and impact on the healthcare system.

#### 3.2.2. Regulatory Pathway for Technologies with Available Comparators

For technologies with existing therapeutic alternatives available approved in the country, pricing decisions should be directly informed by the PII score, which reflects the degree of clinical and methodological innovation demonstrated by the new product. Three pricing pathways are proposed according to the PII classification:

- PII ≥ 75: The technology is considered to present high innovativeness. In this case, the pricing process follows the same pathway as that for technologies without comparators, allowing a provisional base price derived from economic modeling and real-world validation.
- PII 25–74: The technology demonstrates moderate innovativeness. The entry price should initially be benchmarked to the cost of the existing therapeutic alternative. A price premium may be requested if incremental benefits are demonstrated in specific dimensions such as safety, adherence, or convenience.
- PII < 25: The technology exhibits low innovativeness and should enter the market at a price lower than the comparator, reflecting its limited contribution to therapeutic advancement.

For intermediate cases (PII 25–74), manufacturers may apply for a conditional price premium, provided that the request is supported by robust technical evidence. Incremental benefits must be documented across four key dimensions: (1) improved adherence or persistence; (2) enhanced convenience or route of administration; (3) superior safety profile; and (4) alignment with national science and technology policies, such as local production or domestic innovation incentives.

Any granted premium will be temporary and subject to mandatory reassessment two to three years after market entry, based on real-world data collected in Brazil. If the observed outcomes confirm the expected benefits, the premium may be maintained; otherwise, the price will be adjusted downward to ensure consistency between the technology’s actual impact and its economic recognition.

This structured approach strengthens transparency and predictability, ensuring that prices reflect the real and verifiable value of technologies over time while maintaining incentives for genuine innovation and safeguarding the financial sustainability of the healthcare system.

#### 3.2.3. Price Reassessment and Sustainability

For technologies approved under provisional or value-based pricing, the model establishes a mandatory price reassessment two to three years after market entry. Real-World Evidence (RWE) is expected to be generated through post-marketing studies conducted by manufacturers in Brazil, in accordance with CMED and Anvisa requirements. Data collection should include real-world effectiveness, safety outcomes, and economic impact, using observational cohorts or registry-based designs. Study protocols must be submitted for regulatory approval, and results should be made publicly available. These RWE data will serve as the basis for confirming, maintaining, or adjusting the initially approved prices, ensuring that pricing remains aligned with the verified clinical and economic value of the technology.

If real-world evidence confirms the expected benefits, the established price may be maintained. Conversely, if outcomes diverge or fail to demonstrate the anticipated impact, the price should be readjusted downward to ensure alignment with the verified therapeutic and economic value. This mechanism enhances transparency, accountability, and predictability in the regulatory process, reinforcing the link between pricing and value over time and supporting the financial sustainability of the healthcare system.

## 4. Discussion

International experiences demonstrate how the assessment of innovation and therapeutic value has informed pricing and reimbursement decisions in mature regulatory systems [9]. These frameworks served as a conceptual foundation for proposing a new model tailored to the Brazilian context. This study examined the feasibility of applying the Pharmaceutical Innovativeness Index (PII) as a tool to support value-based drug pricing regulation in Brazil. The findings demonstrate that the index’s clinical domains—ATV and UTN—were consistently regarded by experts as central to assessing pharmaceutical innovation. Methodological domains, including SD and MQ, were also valued for ensuring rigor and reducing bias in innovation assessment. Together, these dimensions provide a structured and transparent basis for evaluating the degree of innovativeness of new medicines through a quantitative score, promoting greater reproducibility in regulatory decision-making.

Several implications emerged from the qualitative findings. Participants highlighted dimensions beyond the four core PII domains that could enrich innovation assessment. Safety emerged as a key theme, emphasizing that tolerability improvements may offer substantial clinical and economic benefits even when efficacy remains unchanged. Similarly, incremental innovation—including modifications in formulation, dosing, or administration route—was recognized as relevant for adherence and real-world effectiveness, though it is often undervalued in conventional regulatory frameworks. In fact, formulation changes can enhance therapeutic efficacy or safety, for instance by improving drug dissolution or bioavailability, enabling alternative administration routes that bypass first-pass metabolism, or reducing local irritation through controlled-release or gastro-resistant technologies. Such advances, while sometimes perceived as minor, may translate into improved adherence and better outcomes in practice. Acknowledging these formulation-driven benefits within pricing frameworks could therefore incentivize innovations that yield tangible real-world value, even when their incremental clinical effect size is modest.

Participants also highlighted the strategic importance of fostering national innovation, suggesting that regulatory mechanisms could provide targeted incentives for locally developed technologies. Although broader industrial policies—such as tax benefits or R&D subsidies—remain more common instruments, aligning pricing policy with innovation and industrial development goals could strengthen the domestic pharmaceutical sector and reduce dependency on imported technologies.

From a regulatory perspective, the implementation of VBP strategies in Brazil faces substantial institutional and structural barriers. The National Committee for Health Technology Incorporation (Conitec) conducts post-market evaluations of efficacy, safety, cost-effectiveness, and budget impact to support incorporation into the public health system. However, these assessments occur after CMED has already established the market entry price, creating a disconnect between clinical value appraisal and economic regulation. Moreover, Law No. 14.454/2022 [18], which mandates private sector coverage based on Conitec recommendations, has widened access through private health plans but inadvertently reinforced inequalities within the public system, where operational and financial constraints delay access. This fragmentation between CMED, Conitec, and the Unified Health System (SUS) undermines the coherence of pricing, reimbursement, and coverage decisions, leading to misaligned incentives and limited recognition of true therapeutic value [11].

By integrating the PII into Brazil’s economic regulation framework, it may be possible to bridge this gap between market entry pricing and HTA. The PII introduces explicit, evidence-based criteria that link innovation to price, offering a common evaluative foundation for both CMED and Conitec. To be effective, however, its implementation would require institutional coordination, the establishment of clear methodological guidelines, and a mechanism for periodic price reassessment informed by real-world evidence. This alignment would bring Brazil closer to international best practices, where therapeutic benefit directly informs both pricing and reimbursement.

Importantly, the integration between CMED and Conitec does not require the creation of a new procedural workflow. Once market entry prices are established based on therapeutic value, these can serve as a transparent and consistent benchmark for subsequent discussions and negotiations within Conitec, aligning economic and clinical perspectives from the outset. In the private sector, this same mechanism could address a longstanding regulatory limitation: as ANS does not negotiate drug prices, value-based entry pricing would provide private payers with a clear, evidence-informed reference grounded in innovation and therapeutic benefit. This approach harmonizes decision-making across the public and private spheres, improving coherence and equity in access to high-cost therapies.

The gradual implementation of this model could be achieved through regulatory adaptation of CMED Resolution No. 2/2004 [19], introducing explicit criteria related to therapeutic benefit and innovation in the price-setting process. In parallel, enhanced interagency collaboration and data-sharing mechanisms between CMED, Conitec, and ANS would be essential to ensure consistency between pricing and reimbursement decisions. Practical challenges—such as limited real-world data availability, potential industry resistance, and the need for institutional capacity-building—represent foreseeable barriers but also opportunities for strengthening Brazil’s evidence-based governance in pharmaceutical regulation.

The integration of EF with the PII offers a complementary strategy to address this situation. Using the PII score on the *x*-axis as a proxy for innovativeness allows the construction of an equitable comparison framework across technologies of similar nature (e.g., gene therapies or orphan drugs), anchoring economic modeling on transparent and consistent innovation metrics. This approach can support decision-making in contexts of high uncertainty and limited evidence, where traditional cost-effectiveness thresholds may not be directly applicable.

Despite its promise, the PII should be regarded as a pilot exploratory tool. Its value lies in complementing existing HTA instruments, providing regulators with a structured, reproducible, and transparent mechanism to appraise innovativeness. Future studies should test its applicability across therapeutic areas, refine its weighting structure according to local priorities, and evaluate inter-rater reliability and correlation with established frameworks.

The study has several limitations that must be acknowledged. First, the PII is inherently restricted to innovative medicines, whether chemical or biological, assessed at market entry. It does not extend to generic, similar, or biosimilar drugs, which follow equivalence-based pricing rules in Brazil, nor to medical devices, whose prices are not regulated by CMED. Adapting the framework to these categories would require specific methodological and regulatory adjustments to reflect their distinct evaluation processes. Second, the focus group included a limited number of participants and relied on a qualitative approach, which, while suitable for exploratory analysis, may not capture the full spectrum of stakeholder perspectives. Finally, the application of the proposed pricing model was illustrated through hypothetical case studies, which, although useful for demonstrating feasibility, require empirical validation with real-world data. Additional limitations include the hypothetical nature of the case studies, the subjective nature of expert perceptions, and the absence of empirical validation of the PII’s predictive performance. Future pilot applications across therapeutic classes are warranted to assess the framework’s reproducibility and policy relevance. These boundaries reflect the intended scope of the index rather than conceptual weaknesses, emphasizing that the PII should be viewed as an evolving methodological tool for valuing pharmaceutical innovation within pricing regulation.

## 5. Conclusions

Balancing innovation and cost control in pharmaceutical regulation requires transparent, evidence-based methodologies supported by clear criteria for assessing both incremental and radical innovations. While disruptive innovations may justify higher prices, these must remain proportional to demonstrated clinical benefits and validated through post-marketing evidence. Complementary public investment in R&D and support for incremental innovations can further promote sustainable and equitable access.

The PII provides a structured framework that combines clinical dimensions—Added Therapeutic Value and Unmet Therapeutic Need—with methodological criteria for study design and quality. Experts viewed the PII as a promising tool to improve transparency and consistency in pricing decisions, yet its scope is limited to innovative medicines evaluated at market entry and not intended for equivalence-based categories such as generics, biosimilars, or medical devices. It should therefore be regarded as an exploratory pilot instrument rather than a definitive regulatory solution. Further empirical validation across therapeutic areas, including reproducibility, stakeholder testing, and comparison with established international frameworks, is necessary to confirm its robustness and transferability.

The proposed pricing model demonstrates how the PII could be integrated into Brazil’s regulatory framework in alignment with international value-based practices, linking innovativeness assessment to economic modeling and real-world evidence. By rewarding meaningful innovation while ensuring financial sustainability, this approach outlines a feasible pathway for gradual regulatory reform, provided that its methodological refinement, institutional coordination, and regulatory adaptation are achieved before large-scale implementation.

## Figures and Tables

**Table 1:** Relevance of PII Domains in the Assessment of Innovativeness in the Context of Economic Regulation, According to Focus Group Participants.

	Added Therapeutic Value	Unmet Therapeutic Needs	Study Design	Methodological Quality	Others
Which domains should be considered in the innovativeness analysis?					
Scale from 0 to 10 (mean)	9.26	8.41	7.67	8.00	5.30
sd	1.58	1.93	2.20	2.37	4.03
Relevance of each domain to innovativeness in the context of economic regulation.					
Distribution of 100 points (mean)	33.20	29.00	18.00	19.80	-
sd	9.12	10.21	6.92	9.07	-

sd: standard deviation. Note: Domain relevance scores were normalized to a total of 100 points and used as weighting factors for the four PII domains in the case study analyses (Appendix B.1 and Appendix B.2).

## Data Availability

The original contributions presented in this study are included in the article. Further inquiries can be directed to the corresponding authors.

## Abbreviations

The following abbreviations are used in this manuscript:

AIFA	Agenzia Italiana del Farmaco
ANS	National Supplementary Health Agency (Agência Nacional de Saúde Suplementar)
ASMR	Amélioration du Service Médical Rendu
ATV	Added Therapeutic Value
CMED	Drug Market Regulation Chamber (Câmara de Regulação do Mercado de Medicamentos)
Conitec	National Committee for Health Technology Incorporation (Comissão Nacional de Incorporação de Tecnologias no SUS)
EF	Efficiency frontier
HAS	Haute Autorité de Santé
HTA	Health Technology Assessment
MQ	Methodological Quality
PII	Pharmaceutical Innovativeness Index
R&D	Research and Development
SD	Study Design
SUS	Brazilian Unified Health System (Sistema Único de Saúde)
UTN	Unmet Therapeutic Need
VBP	Value-Based Pricing

## Appendix A

The Pharmaceutical Innovativeness Index (PII) is a structured, transparent, and reproducible tool designed to quantify the degree of innovativeness of new medicines at market entry. It provides a numerical score ranging from 0 to 100, allowing for a standardized assessment of innovation based on both therapeutic relevance and evidence robustness. The PII can be applied to any therapeutic area by systematically evaluating clinical data from pivotal trials and complementary real-world evidence. The resulting score classifies innovations as high, moderate, or low, supporting consistent and transparent pricing and regulatory decisions [13].

The index comprises four domains, divided into clinical and methodological dimensions:

- Added Therapeutic Value (ATV)—measures the magnitude of clinical benefit compared with the current standard of care.
- Unmet Therapeutic Need (UTN)—evaluates the extent to which the new technology addresses an unfulfilled medical need.
- Methodological Quality (MQ)—assesses the internal validity of pivotal trials, including randomization, blinding, sample size, and risk of bias.
- Study Design (SD)—considers the external validity and adequacy of the comparator used, prioritizing active-controlled trials over placebo-controlled ones when alternatives exist.

Each domain was assigned a weight that reflects its relative importance in determining innovativeness: ATV (35%), UTN (25%), MQ (20%), and SD (20%) [13]. However, these weights may be adapted to reflect local preferences or policy priorities, depending on the decision-making context and therapeutic area. Within each domain, the total weight is distributed across pre-established levels, each corresponding to a qualitative classification that is translated into a quantitative score.

For ATV and UTN, five hierarchical levels are defined, and for MQ and SD, three levels are defined. Each level receives a numerical score, and the final PII score corresponds to the sum of the weighted points across all domains in which the technology is classified. This structure allows flexibility while maintaining methodological consistency, ensuring that the PII can be tailored to local regulatory frameworks or adapted to specific therapeutic areas and outcomes of interest [13].

## Appendix B

### Appendix B.1. Use Case 1—Pricing a Technology with Available Comparator

In this hypothetical case, a new oncology drug (MedOnco, fictitious name) was submitted for approval in Brazil for the treatment of metastatic cancer with a specific target mutation in patients previously treated with cytotoxic chemotherapy. At the time of submission, the main treatment available in Brazil for this indication was a chemotherapeutic agent approved the previous year and used as the standard of care.

PII and Innovativeness Category

The PII was applied to assess MedOnco’s degree of innovation and determine its maximum entry price according to the proposed regulatory model. MedOnco scored 58.9 points, indicating moderate innovativeness and qualifying for pricing based on the available comparator.

- UTN: Important—chemotherapy was the only available option but associated with high toxicity, including febrile neutropenia, renal failure, and cardiotoxicity. MedOnco offered a less toxic alternative, though without significant improvement in overall survival.
- ATV: Poor—MedOnco increased median survival by 15.8 months versus placebo (20.2% reduction in mortality risk) but did not show superiority over chemotherapy.
- SD: Partially adequate—clinical trials used a placebo comparator despite the availability of active treatment, limiting evaluation of incremental benefit.
- MQ: High—low risk of bias according to the Cochrane Risk of Bias 2.0 tool.

**Table A1:** Domain-level PII Scoring and Weighting for Use Case 1.

	Level	Weight	Points
UTN	Important	29.0	21.75
ATV	Poor	33.2	8.30
SD	Partially adequate	18.0	9.00
MQ	Low risk of bias	19.8	19.80
Final score			58.9

Legend: Added Therapeutic Value (ATV) and Unmet Therapeutic Need (UTN)—and two methodological—Methodological Quality (MQ) and Study Design (SD). Note: The domain weights were established based on the relevance attributed by focus group participants, as shown in Table 1.

Base Price Determination

With a PII score of 58.9 (between >50 and ≤75) and an existing therapeutic alternative, MedOnco was classified in the “at comparator price” category. The base price was aligned with the annual cost of chemotherapy (R$174,588.10), corresponding to a monthly price of R$14,549.00.

Incremental Benefit Analysis

A 9% price premium was granted based on two incremental benefit dimensions:

- Safety (4%)—lower incidence of severe adverse events, though long-term cardiovascular safety remained uncertain.
- Convenience (5%)—oral administration compared with intravenous chemotherapy.

The final adjusted monthly price for MedOnco was R$15,858.41.

Real-World Evidence (RWE) Reassessment

To maintain the incremental benefit premium, the manufacturer committed to conducting RWE studies in Brazil to validate the claimed benefits, including reduced adverse events and improved adherence due to oral administration. These data must be submitted within two to three years for CMED reassessment. If benefits are not confirmed, the price may be adjusted to reflect the actual clinical and economic impact.

### Appendix B.2. Use Case 2—Pricing of Orphan and Advanced Therapies

In this hypothetical case, a biotechnology company submitted a gene therapy (TGen, fictitious name) for approval in Brazil for the treatment of Severe Congenital Enzyme Deficiency (SCED), a rare and progressive genetic disorder with no available therapies other than best supportive care. Patients with SCED experience progressive multisystem failure, frequent hospitalizations, dependence on ventilatory and nutritional support, and high mortality before age five.

TGen is a one-time gene therapy designed to introduce a functional gene to correct the underlying genetic defect. International trials demonstrated improvements in enzyme activity and event-free survival, with a reduced need for hospitalizations and intensive care.

As no therapeutic alternatives were available, TGen followed the regulatory model for technologies without comparators, allowing a provisional price based on economic modeling, subject to the following conditions:

- Preliminary evaluation using the PII;
- Price justification through cost-effectiveness and efficiency frontier analysis;
- Incorporation of Brazilian supportive care cost data;
- Modeling limited to the clinical trial follow-up period;
- Commitment to price reassessment within two to three years based on real-world evidence.

PII and Innovativeness Category

TGen was assessed using the PII with the following results:

- UTN: Maximum—no specific treatment options beyond supportive care.
- ATV: Low—improved enzyme function but no confirmed gains in overall survival or quality of life.
- SD: Inadequate—single-arm trial; in the absence of active comparators, historical or supportive care controls would have been preferable.
- MQ: High—low risk of bias despite design limitations, as assessed using appropriate methodology.

**Table A2:** Domain-level PII Scoring and Weighting for Use Case 2.

	Level	Weight	Points
UTN	Maximum	29.0	29.00
ATV	Poor	33.2	8.30
SD	Inadequate	18.0	0.00
MQ	Low risk of bias	19.8	19.80
Final score			57.1

Legend: Added Therapeutic Value (ATV) and Unmet Therapeutic Need (UTN)—and two methodological—Methodological Quality (MQ) and Study Design (SD). Note: The domain weights were established based on the relevance attributed by focus group participants, as shown in Table 1.

Base Price Determination

Although the PII score was 57.1, TGen was considered clinically relevant as the only available therapy for SCED. The manufacturer proposed an initial price of R$2,450,000.00 per patient, justified by:

- Cost-effectiveness modeling showing substantial reductions in hospitalizations, ventilatory support, and emergency interventions;
- Efficiency frontier analysis using the cost of supportive care, which exceeds R$800,000.00 annually due to frequent ICU admissions and complex management.

Real-World Evidence (RWE) Reassessment

To maintain the approved price, the company committed to submitting an updated dossier with RWE within two to three years, including:

- Post-marketing clinical data confirming survival and reduction in severe events;
- Brazilian cost-of-illness studies comparing TGen-treated patients with those receiving supportive care;
- Revised economic modeling incorporating long-term data.

If projected clinical and economic benefits are not confirmed, the price may be adjusted accordingly to reflect the therapy’s actual value, ensuring sustainable financing within the healthcare system.
